# Fertility and sexual activity in patients with Triple A syndrome

**DOI:** 10.3389/fendo.2024.1357084

**Published:** 2024-03-13

**Authors:** Katja K. Dumic, Claudine Heinrichs, Katrin Koehler, Angela Huebner, Miroslav Dumic, Vesna Kusec, Tina Dusek, Friederike Quitter

**Affiliations:** ^1^ Department of Paediatric Endocrinology and Diabetes, University Hospital Centre Zagreb, University of Zagreb, School of Medicine, Zagreb, Croatia; ^2^ Université libre de Bruxelles (ULB), Hôpital Universitaire de Bruxelles (H.U.B), Hôpital Universitaire des Enfants Reine Fabiola (HUDERF) Paediatric Endocrinology Unit, Bruxelles, Belgium; ^3^ Department of Pediatrics, Faculty of Medicine and University Hospital Carl Gustav Carus, Technische Universität Dresden, Dresden, Germany; ^4^ Department of Laboratory Medicine, University Hospital Centre Zagreb, University of Zagreb, School of Medicine, Zagreb, Croatia; ^5^ Department of Endocrinology and Diabetes, University Hospital Centre Zagreb, University of Zagreb, School of Medicine, Zagreb, Croatia

**Keywords:** Allgrove syndrome, erectile dysfunction, ejaculatory dysfunction, pregnancy, sexual activity

## Abstract

**Objective:**

Triple A syndrome, caused by autosomal recessively inherited mutations in the *AAAS* gene is characterized by alacrima, achalasia, adrenal insufficiency, and neurological impairment. To the best of our knowledge, no patients of both sexes have been reported to have offspring. Our aim was to assess the causes of infertility in male patients with this multisystemic syndrome, and to present a female patient that spontaneously conceived a child.

**Design:**

Cross-sectional study.

**Methods:**

Six males aged 19-48 years were included. Gonadotropins, testosterone, DHEAS, androstenedione, inhibin B, anti-Mullerian hormone measurements and testicular ultrasound were performed.

**Results:**

All six male patients had impaired general health and neurological symptoms including erectile and ejaculatory dysfunction. None of them had an offspring. The only demonstrated cause of infertility in our male patients was erectile and ejaculatory dysfunction which precludes sexual intercourse. Our patients had normal libido but were sexually abstinent. Except for low adrenal androgen levels, the concentrations of all measured hormones as well as testicular ultrasound were normal which may indicate the possibility of spermatogenesis in male patients with triple A syndrome. Little is known about fertility in female patients, but based on our observations spontaneous pregnancies seem to be possible.

**Conclusion:**

Our results contribute to still scarce knowledge on fertility in patients with Triple A syndrome and as well represents a foundation for further research on causes of infertility and possible treatment options.

## 1 Introduction

Triple A syndrome (Allgrove syndrome, OMIM #231550) is caused by autosomal recessively inherited mutations in the *AAAS* gene on chromosome 12q13 encoding the nuclear pore complex (NPC) protein ALADIN (1, 2). Nuclear pore proteins form a channel within the nuclear membrane enabling nucleocytoplasmic transport of proteins and RNA and therefore play a role in some key cellular processes such as cell growth and differentiation, gene expression and DNA repair (3). Mutations in the *AAAS* gene lead to mis-localization of mutated ALADIN proteins to the cytoplasm and therefore NPC dysfunction (4). This results in increased cellular accumulation of reactive oxygen species. Several studies demonstrated increased susceptibility to oxidative stress in ALADIN-deficient adrenal cells and patient fibroblasts (5–8).

Triple A syndrome is a multisystemic disorder which is characterized by achalasia, alacrima, ACTH-resistant adrenal insufficiency, and neurological impairment (9, 10).

To the best of our knowledge no patients with triple A syndrome, regardless of sex, have been reported having offspring. So far, there are no studies on reproductive function in patients with this progressive multisystemic disease.

The objective of this study was to investigate factors that could influence reproductive function in a cohort of six adult male patients with triple A syndrome. Moreover, we report on a child of a female triple A patient with a homozygous *AAAS* mutation with a consanguineous father who is heterozygous for the identical *AAAS* mutation.

## 2 Methods

Genomic DNA was purified from peripheral blood for DNA analysis using the QIAamp DNA Blood Mini Kit (Qiagen, Hilden, Germany). Primer sequences and PCR conditions used for amplification of the 16 exons of *AAAS* gene including exon–intron boundaries are available upon request. Sequencing was performed using the BigDye Terminator v1.l. Cycle Sequencing Kit and an ABI 3130xl Genetic Analyzer (Applied Biosystem, Foster City, CA, USA).

Testicular ultrasound was performed by one examiner using GE Healthcare LOGIO S8 and E9 ultrasound system Aloca scanner equipped with 15-MHz linear transducer probe.

Sialometry and tests for oral candidiasis were performed as previously described (11).

Blood was drawn after an 8-10 h overnight fast (2 h after morning medication). Sera were separated and tests were performed immediately or samples were frozen at -20°C until the assay was performed. Standard recommended biochemical methods for all variables were used. Hormone measurements were performed on automated or semi-automated platforms by using commercial kits: luteinizing hormone (LH), follicle-stimulating hormone (FSH), and testosterone (T) by Abbott Laboratories (USA); prolactin, dehydroepiandrosterone sulphate (DHEAS), androstenedione, anti-Mullerian hormone (AMH), and inhibin B.

Structured interviews about the sexual function and habits were performed in all patients.

The study was approved by the Ethics Committee of University Hospital Zagreb, Zagreb, Croatia. Written informed consent for the study was obtained from all patients.

## 3 Patients

The study included six male patients from five different non-consanguineous families of Croatian origin. Two symptoms and clinical course of the brothers, patients no. 1 and no. 2, as well as from patients no. 3, 4, and 5 were previously described (11–13).

### 3.1 Patient no. 6

This 24-year-old male is the only child of healthy non-consanguineous parents. At the age of one year, bilateral vesicoureteral reflux grade IV, hydroureteronephrosis and urosepsis were diagnosed. At the age of two years, a bilateral antireflux operation was performed. As progressive hydroureteronephrosis, repeated uroinfections, renal insufficiency, incontinence, and neurogenic urinary bladder developed, at the age of five years a bilateral antireflux operation was repeated. From the age of five until the age of ten years urine derivation has been managed through bilaterally placed ureterostomies. Since then, the patient performs self-catheterisation during the day and a permanent catheter during nighttime. Due to repeated urosepsis episodes, the patient developed chronic renal insufficiency, requiring dialysis on several occasions.

At the age of three years, he developed various neurological symptoms such as hyperreflexia, calves hypotrophy, pes cavus, and gait disturbance. In view of these symptoms hereditary polyneuropathy, similar to Charcot-Marie-Tooth disease was diagnosed.

At the age of seven years, the patient developed hyperpigmentation, fatigue, exhaustion, and dysphagia. At this time his height was 123 cm (-0.26 SD), while his weight was 19.5 kg (-1.5 SDS), and BMI 12.9 kg/m^2^ (-2.1 SDS). He had cutis anserina, palmoplantar hyperkeratosis, mouth dryness, glossitis, fungiform papillae of the tongue, and dental caries. Salivation was deficient (0.1 mL/5 min), and the test for oral candidiasis was positive. Neurological examination revealed brisk tendon reflexes and weakened ankle jerks. Babinski sign was negative. Electromyography (EMG) and nerve conduction studies revealed mixed motor and sensory demyelinating neuropathy in lower and upper extremities, and decreased conduction velocities. Muscle strength was 4/5 (according to Medical Research Council (MRC) scale) in all four extremities. The speech was nasal. Cognitive development was normal. Schirmer test confirmed decreased tear production (2 mm after 1 and 5 min). Fundoscopy showed no abnormalities. Laboratory investigation revealed elevated plasma ACTH (greater than 2,000 pmol/L, n.r. basal 2 to 11 pmol/L), low cortisol (5 nmol/L, n.r. basal morning 140 to 690 nmol/L), undetectable DHEAS and androstenedione, and normal levels of aldosterone and PRA.

Molecular genetic analysis confirmed the diagnosis of triple A syndrome caused by compound heterozygous mutation in the *AAAS* gene consisting of missense mutation c.887C>A (p.S296Y) on one allele and nonsense mutation c.1159C>T (p.Q387X) on the other allele. The father is heterozygous carrier of the p.Q387X mutation and the mother is heterozygous carrier of the p.S296Y mutation.

Patient treatment with hydrocortisone, 15 mg/m^2^/day, artificial tears and saliva, and antimycotics were introduced at the age of 7 years.

During the course of follow-up over 16 years deterioration of neurological symptoms and some new manifestations developed, including pronounced muscle weakness and atrophy (predominantly of the calves and hypothenars). Furthermore, pes cavus and the foot and talocrural contractures caused severe walking difficulties, so that the patient is to undergo achillotenoplastic and corrective osteotomy. Postural hypotension, osteoporosis and swallowing difficulties were recognized. Pubertal development was normal. At the age of 24 years he had a testicular volume of 16 and 15 mL, respectively. The patient has normal libido, but suffers from erectile and ejaculatory dysfunction the extent of which is difficult to determine because of the usage of a urinary catheter. At the age of 24 years he has no partner.

### 3.2 Female patient

The female patient outside the study of the males was previously described (13). In brief, this now 44-year-old female with the full spectrum of triple A syndrome was diagnosed at the age of 9.5 years. She presented the typical features of alacrima, achalasia, axonal sensorimotor polyneuropathy and primary adrenal insufficiency due to a homozygous mutation c.1024 C>T, p.Arg342* in the *AAAS-*gene. Her onset of puberty was at the age of 11^9/12^ years with thelarche whereas menarche was observed at the age of 16^7/12^ years. She underwent Nissen surgery (cardiomyotomy) at the age of 16 years. She was aware about the genetics aspects of her triple A disease, however genetic counseling was refused. At the age of 29 years she got pregnant. Despite a maternal height of 144.2 cm, pregnancy, labor and delivery proceeded normally. Genetic analyses were performed on the patient and her husband, and later on the child.

## 4 Results

Clinical and biochemical data, results of molecular genetic analysis, and testicular ultrasound examination for six male patients with triple A syndrome are summarized in **Table 1**.

**Table 1:** Characteristics of six male patients with triple A syndrome.

Patient	1	2	3	4	5	6
**Age** (years)	48	42	19	32	38	24
**Tanner stage**	5	5	5	5	5	5
***AAAS* gene mutation**	c.43C>A(p.G14fs) rs121918549/c.787T>C (p.S263P) rs121918550	c.43C>A(p.G14fs) rs121918549/c.787T>C (p.S263P) rs121918550	c.787T>C (p.S263P) rs121918550 /c.887C>A (p.S296Y) rs1450008394	c.43C>A(p.G14fs) rs121918549/c.1159C>T (p.Q387X) rs763820204	c.1159C>T (p.Q387X) rs763820204/c.1159C>T (p.Q387X) rs763820204	c.887C>A (p.S296Y) rs1450008394/c.1159C>T (p.Q387X) rs763820204
**FSH** (n.r. 1.4-7.5 U/L)	3.6	2.0	2.7	3.1	7.3	6.2
**LH** (n.r. 1.5 - 6.30 U/L)	1.5	2.2	2.9	5.7	2.7	6.8
**TSH** (n.r. 0.6 - 3.8 mIJ/L)	2.4	3.0	1.8	2.7	2.5	2.2
**Testosterone** (n.r. 12.1 - 28.5 nmol/L)	11.6	18.4	18.1	12.7	16.5	22.2
**DHEAS** (n.r. 3.5 - 14 µmol/L)	<0.1	0.1	0.2	0.7	0.5	0.3
**Androstenedione** (n.r. 1.8 - 7.8 nmol/L)	0.9	<0.1	0.1	0.3	0.9	0.2
**AMH** (n.r. 13 - 90 pmol/L)	40.7	42.4	67.4	51.0	43.6	31.3
**Inhibin B** (n.r. 95 - 323 ng/mL)	296	280	116	143	96	141
Testicular volume ^*^ **L/R** (mL)	15/14	12/13	16/15	14/15	12/13	16/15

FSH, Follicle Stimulating Hormone; LH, Luteinizing Hormone; TSH, Thyroid Stimulating Hormone; DHEAS, Dehydroepiandrosterone Sulfate: AMH, Anti-Müllerian Hormone; n.r, normal range; L, left; R, right.

Testi.

^*^ Testicular volume was assessed by ultrasound, using the following formula: V (mL) = length (cm) x width (cm) x depth (cm) x 0.52 (14). Results were round up to whole number.

All six male patients (no. 1-6) had normal pubertal development, genital appearance, testicular size, and testes of normal homogeneous echotexture. Normal plasma levels of gonadotropins, testosterone, inhibin B, AMH, cortisol (under treatment with hydrocortisone), and low-levels of adrenal androgens were detected. Their libido was normal, but all had erectile and ejaculatory dysfunction and never had sexual intercourse. Only the youngest patient no. 3 registered weak morning erections, whose frequency decreased. Over the time this patient found it increasingly difficult to achieve erection. Other patients recall how they registered occasional nocturnal and daily erections in adolescence, but by entering adulthood, they generally find it difficult to achieve. The oldest patients of our series, patients no. 1, 2 and 5, progressively developed complete erectile dysfunction, which is consistent with progressive course of neurological impairment including dysfunction of the autonomous nervous system in this multisystemic disease.

Four patients (no. 1, 2, 4 and 5) tried therapy with 50 mg phosphodiesterase type 5 inhibitor sildenafil citrate (Viagra), but the effect on erections was weak, and none of the patients were able to ejaculate. Only one patient (no. 1) is married, but did not plan biological fatherhood or to adopt a child due to severely impaired general health. The remaining five patients do not have a sexual partner. All six adult patients have severe neurological deficits. Patient no. 6 additionally has severe renal insufficiency and wears a permanent urinary catheter as described. Patient no. 4, with numerous disabling neurologic manifestations, died in adrenal crisis at the age of 34 years.

Discussing fertility in triple A patients, we additionally report a female triple A patient who conceived and delivered a baby. The patient was diagnosed with triple A syndrome at the age of 9.5 years, in the context of hypoglycemia and seizures (13). Her husband is a first cousin and heterozygous for the identical family mutation in the *AAAS-*gene.

The couple desired to have children, and the patient conceived at the age of 29 years with an uneventful course of the pregnancy. Preconceptional genetic consultation for the couple could not be organized, because she was getting rapidly pregnant two months after marriage. She and her husband were not keen on a prenatal diagnosis, and were opposed to any intervention of pregnancy, for religious reasons. The girl was born with 40 + 1 weeks of gestation with normal body measures (birth weight 3120 g, length 49.5 cm, head circumference 35 cm), the child’s birth was assisted with a vacuum extractor. Due to respiratory distress ventilation with a mask for a few minutes after birth was necessary (Apgar score 7/7/8). The child was vital after birth and clinical examination was unremarkable. On the first day of life cortisol (102.08 nmol/L µg/dL) and ACTH (4.9 pmol/L) were measured within normal ranges. Synacthen test was performed at the third day of life and showed a normal peak cortisol of 849.71 nmol/L. No hypoglycemias were observed. Postnatal genetic investigation revealed the same homozygous mutation in exon 11 (c.1024 C>T, p. Arg342*) in the *AAAS-*gene. Hydrocortisone substitution was started at the age of five years due to a pathological Synacthen test as well as mineralocorticoid substitution with fludrocortisone. Additionally, she suffered from achalasia, alacrimia and the neurological conditions dyslexia and dyscalculia. The girl had onset of puberty with thelarche at the age of eleven years and pubarche at the age of 12^4/12^ years.

Expressing a strong wish to have more children, five years after the first pregnancy the mother conceived again, but experienced a miscarriage after 6 weeks. Menstrual cycles were regular every 29 days. MRI scan of the pelvis showed a polyp of the endometrium but otherwise no relevant pathologies. Anti-Mullerian hormone was low with 1.06 pmol/L indicating a low ovarian reserve.

To our knowledge our index case is the first female patient with genetically proven triple A syndrome who conceived and gave birth to a child.

## 5 Discussion

A search of the literature reveals no reports of triple A syndrome patients of either sex having offspring. In addition, there is no data on the desire or attempt of these individuals to become parents, their libido, sexual activity, reproductive function, or conceptions and miscarriages. There is also no data on the investigation or treatment of infertility for these patients. One of the possible reasons for this could be that most of described patients are children or adolescents (2/3 from about 280 cases reported worldwide) and only a few long-term follow-up longitudinal studies exist. A second reason for the low reported reproduction rate could be difficulties for triple A syndrome patients in finding partners due to the various health problems connected with the disorder itself.

The first investigation of a role of ALADIN in fertility was conducted by Huebner et al. on an animal model for human triple A syndrome, producing mice lacking a functional *AAAS* gene (15). They showed that the lack of ALADIN in mice does not cause a severe triple A syndrome phenotype. However, homozygous knockout female mice were infertile whereas males were fertile. Carvalhal et al. revealed that infertility in female mice lacking ALADIN is caused by multiple defects in oocyte maturation disclosing an important role of ALADIN in meiosis (16). Despite these findings in the mouse model, we report a female patient with a homozygous *AAAS* mutation who had an offspring but then experienced a miscarriage. The pregnancy occurred despite the fact that the patient shows the full clinical picture of this serious disorder. The patient’s mutation is a stop mutation that has also been detected in other independent, unrelated patients (n=8 from n=6 further families of our collection of 218 families with *AAAS* mutations; unpublished data). Among these patients six are females. The stop mutation p.Arg342* shorten the C-terminal region of the protein with loosing of potential localization signal sequences. However, stop mutations more proximal or distal also led to similar phenotype. A relationship between the location of the mutation and the severity of the disease could not be established (17). In this study, we did not investigate protein function further. However, in previous research, the mislocalization of mutated ALADIN away from the nuclear pore was noted (18). All disease-causing mutations (stop mutations, frameshift mutations or point mutations) were mainly mislocalized in the cytoplasm or the nucleus (18). Functional studies showed that cells from triple-A patients had higher levels of ROS and were less able to respond to oxidative stress (6).

In our study of fertility in males with triple A syndrome, we show that none of them had offspring. They all entered puberty on time, and their sexual development was appropriate for age. Only one is married, while the others do not have partners. Despite normal libido, none of them have had sexual intercourses due to erectile dysfunction. Treatment attempts with sildenafil were not successful. The erectile dysfunction probably reflects the impairment of the autonomous nervous system which is described in about 30% of triple A patients (19). Additional symptoms can include postural hypotension, cardiac dysrhythmias or unequal pupils (20). Our patients had normal testicular function regarding testosterone production and testicular morphology. Other possible reasons for erectile/ejaculatory disfunction such as: tobacco, alcohol and drug abuse, trauma exposure to infection, radiation or overheating, history of undescended testicles, metabolic syndrome, varicocele were excluded in all patients. We therefore assume that spermatogenesis is probably preserved in this group of patients. Unfortunately, due to the ejaculatory dysfunction, the semen analysis which would confirm our assumption could not be done. Furthermore, due to their medical impairment, the patients declined testicular biopsy.

Sexual inactivity due to complete erectile and ejaculatory dysfunction is obviously an important cause of infertility in our group of patients. In addition, these patients do not show interest in parenting or additional diagnostic procedures. Beside erectile and ejaculatory dysfunction, their seriously impaired overall health restricts the daily activities of these patients and makes it difficult for them to find a partner.

Results of fertility investigation in our small cohort of males with triple A syndrome represents an incentive for further research into the causes and therapy of infertility in patients of both sexes with this multisystemic progressive debilitating disease.

## Data availability statement

The original contributions presented in the study are included in the article/supplementary material, further inquiries can be directed to the corresponding author.

## Ethics statement

The studies involving humans were approved by University Hospital Centre Zagreb. The studies were conducted in accordance with the local legislation and institutional requirements. Written informed consent for participation in this study was provided by the participants’ legal guardians/next of kin. Written informed consent was obtained from the individual(s) for the publication of any potentially identifiable images or data included in this article.

## Author contributions

KD: Conceptualization, Investigation, Writing – original draft, Writing – review & editing. CH: Writing – original draft, Writing – review & editing, Conceptualization, Formal analysis. KK: Conceptualization, Writing – original draft, Writing – review & editing. AH: Writing – original draft, Writing – review & editing, Conceptualization. MD: Writing – original draft, Writing – review & editing, Supervision. VK: Writing – original draft, Writing – review & editing, Data curation, Formal analysis, Validation. TD: Writing – original draft, Writing – review & editing, Investigation, Methodology. FQ: Writing – original draft, Writing – review & editing, Data curation.
